# Manipulation of environmental oxygen modifies reactive oxygen and nitrogen species generation during myogenesis

**DOI:** 10.1016/j.redox.2016.01.011

**Published:** 2016-01-21

**Authors:** Rachel McCormick, Timothy Pearson, Aphrodite Vasilaki

**Affiliations:** MRC-Arthritis Research UK Centre for Integrated Research into Musculoskeletal Ageing, Department of Musculoskeletal Biology, Institute of Ageing and Chronic Disease, University of Liverpool, Liverpool L7 8TX, UK

**Keywords:** CuZnSOD, copper, zinc superoxide dismutase, MnSOD, manganese superoxide dismutase, mpc's, myogenic precursor cells, NFκB, nuclear transcription factor κappa B, NO, nitric oxide, ROS, reactive oxygen species, RONS, reactive oxygen and nitrogen species, SOD, superoxide dismutase, SOD1, copper, zinc superoxide dismutase, WT, wild type, H_2_O_2_, hydrogen peroxide, Reactive oxygen and nitrogen species, Low oxygen concentration, Skeletal muscle, Antioxidant defence enzymes

## Abstract

Regulated changes in reactive oxygen and nitrogen species (RONS) activities are important in maintaining the normal sequence and development of myogenesis. Both excessive formation and reduction in RONS have been shown to affect muscle differentiation in a negative way. Cultured cells are typically grown in 20% O_2_ but this is not an appropriate physiological concentration for a number of cell types, including skeletal muscle. The aim was to examine the generation of RONS in cultured skeletal muscle cells under a physiological oxygen concentration condition (6% O_2_) and determine the effect on muscle myogenesis.

Primary mouse satellite cells were grown in 20% or 6% O_2_ environments and RONS activity was measured at different stages of myogenesis by real-time fluorescent microscopy using fluorescent probes with different specificities i.e. dihydroethidium (DHE), 4-amino-5-methylamino-2′,7′-difluorofluorescein diacetate (DAF-FM DA) and 5-(and-6)-chloromethyl-2′,7′ -dichlorodihydrofluorescein diacetate (CM-DCFH-DA).

Data demonstrate that satellite cell proliferation increased when cells were grown in 6% O_2_ compared with 20% O_2_. Myoblasts grown in 20% O_2_ showed an increase in DCF fluorescence and DHE oxidation compared with myoblasts grown at 6% O_2_. Myotubes grown in 20% O_2_ also showed an increase in DCF and DAF-FM fluorescence and DHE oxidation compared with myotubes grown in 6% O_2_. The catalase and MnSOD contents were also increased in myoblasts and myotubes that were maintained in 20% O_2_ compared with myoblasts and myotubes grown in 6% O_2_. These data indicate that intracellular RONS activities in myoblasts and myotubes at rest are influenced by changes in environmental oxygen concentration and that the increased ROS may influence myogenesis in a negative manner.

## Highlights

• Environmental O_2_ concentrations were found to influence myogenesis *in vitro.*
• Proliferation of primary muscle cells was increased when cells were grown in 6% O_2_.
• Cells grown in 20% O_2_ showed increased RONS formation compared with cells in 6% O_2_.
• Cells grown in 20% O_2_ had higher antioxidant defence enzyme contents.
• These data suggest that RONS generated at 20% O_2_ may limit myogenesis *in vitro*.

## Graphical abstract

**Figure:**
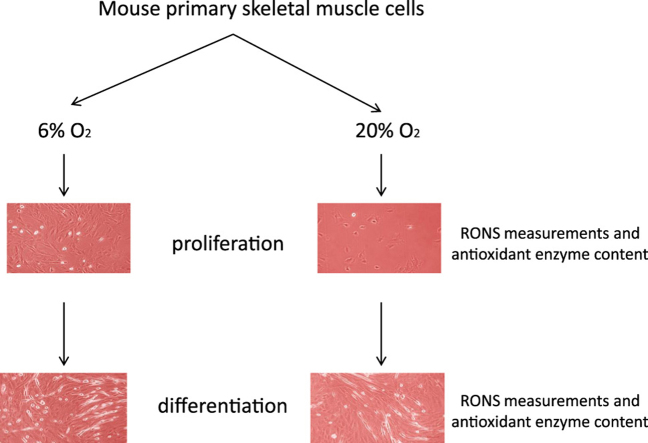

## 1 Introduction

Skeletal muscle satellite cells are ubiquitous mononuclear cells, classically identified by their location between the fibre plasmalemma and basal lamina [1]. Satellite cells play an important role during skeletal muscle injury and regeneration. When skeletal muscle damage occurs, satellite cells within the muscle bulk are activated and proliferate to become myogenic precursor cells (mpc's otherwise known as myoblasts; [2]). Proliferating myoblasts migrate to the damaged region of the muscle, fuse to form myotubes and differentiate to form skeletal muscle. Regeneration of skeletal muscle *in vivo* involves similar processes to those occurring during myogenesis and can be studied in well-characterised cell culture models.

The environmental O_2_ concentration used for satellite cell cultivation *in vitro* is almost always 20%, whereas normal adult skeletal muscle tissue O_2_ levels are significantly lower, potentially between 1.8 and 10.5% [3], [4]. Environmental oxygen concentration has been previously shown to modify satellite cell behaviour [3] in a process that has been linked to reactive oxygen species (ROS) generation [5]. The mechanisms by which ROS mediate myogenesis are unclear but are likely due to changes in gene expression via redox-sensitive transcription factor activation [5]. However, the pattern of generation of specific ROS in skeletal muscle cells during the processes of myogenesis under different oxygen concentrations is currently unknown.

The aim was therefore to examine the activities of RONS in cultured skeletal muscle cells under approximately physiological conditions (6% oxygen) compared with 20% O_2_ and also determine the effect of the different O_2_ concentrations on muscle myogenesis. Primary skeletal muscle cultures were grown in 20% or 6% oxygen environments and RONS were assessed at different stages of myogenesis using RONS-sensitive fluorescent probes [6], [7], [8]. Use of these probes allows the assessment of specific RONS in single cells in real time. The fluorescent probes dihydroethidium (DHE), 5-(and-6)-chloromethyl-2′,7′ -dichlorodihydrofluorescein diacetate (CM-DCFH-DA) and 4-amino-5-methylamino-2′,7′-difluorofluorescein diacetate (DAF-FM DA) were used in this study. DCFH reacts with hydrogen peroxide (H_2_O_2_) in the presence of peroxidases and less rapidly with some other ROS, DAF-FM reacts with NO and peroxynitrite, and DHE is primarily oxidised by superoxide. Changes in fluorescence in skeletal muscle myoblasts and myotubes were measured using fluorescence microscopy.

Our hypothesis was that myoblasts and myotubes grown in 20% O_2_ would have increased superoxide content leading to an increase in intracellular DCF and DHE oxidation but no effect on DAF-FM fluorescence compared with cells grown in 6% O_2_ and that this would be associated with reduced myogenesis in the myoblasts grown in 20% O_2_.

## 2 Materials and methods

### 2.1 Cultures of skeletal muscle myoblasts and myotubes

Myoblasts were derived from adult (4–8 months old) male wild-type (WT) mice. Primary mouse myoblasts were prepared from hind leg muscles as previously described [6]. Briefly, muscles were digested in 0.1% pronase solution. Cells were cultured in 35 mm gelatin coated tissue culture plates in DMEM containing 20% (v/v) FCS. Cells were incubated at 37 °C in a water saturated atmosphere containing 5% (v/v) CO_2_ in either 20% or 6% oxygen environments. To induce myotube formation the medium was replaced with DMEM containing 2% horse serum containing 2% horse serum (HS) with 0.45% (*w*/*v*) glucose with 2 mM glutamine, 50 I.U./ml penicillin and 50 μg/ml streptomycin.

### 2.2 Loading of cells with fluorophores

To detect intracellular ROS and nitric oxide, myoblasts and myotubes at 5–7 days following differentiation (multinuclear fused) were loaded with different fluorophore probes; 5-(and-6)-chloromethyl-2′,7′ -dichlorodihydrofluorescein diacetate (CM-DCFH-DA) (Molecular Probes™, Invitrogen) was used as a general probe for ROS, 4-amino-5-methylamino-2′,7′ -difluorofluorescein diacetate (DAF-FM-DA) as an indicator of nitric oxide and dihydroethidium (DHE) as an indicator of superoxide activity (all purchased from Molecular Probes™, Invitrogen). Cells were loaded with CM-DCFH-DA (10 μM) DHE (5 μM) or DAF-FM-DA (10 μM) in D-PBS for 30 min at 37°. Cells were then washed with D-PBS and the media replaced with D-PBS alone.

### 2.3 Microscopy and fluorescent imaging

Images were obtained using a C1 confocal laser-scanning microscope (Nikon Instruments Europe BV, Surrey, UK) equipped with a 405 nm excitation diode laser, a 488 nm excitation argon laser, and a 543 nm excitation helium-neon laser. Emission fluorescence was detected through a set of 450/35, 515/30 and 605/15-emission filters. Fluorescence images were captured and analysed with the EZC1 V.3.9 (12 bit) acquisition software. For the cultures grown at 6% oxygen, oxygen concentration was maintained at 6% for the duration of the experiments. All experiments were carried out at 25 °C.

### 2.4 Analysis of MnSOD, Cu/ZnSOD and catalase content of myoblasts and myotubes

Myoblasts and myotubes at 5–7 days following differentiation were harvested and sonicated in 1% SDS containing 1 mM iodoacetimide, 1 mM benzithonium chloride, and 5.7 mM phenylmethylsulfonyl fluoride and 5 mM EGTA (Sigma Co.). Following sonication, cellular debris was removed by centrifugation, and samples were stored at −70 °C until analysis. Protein content of samples was determined by using the bicinchoninic acid method (Sigma Co.). Twenty micrograms of total cellular protein was separated on SDS-PAGE followed by Western blotting. Ponceau S staining (Po-S; Sigma Co.) was used to visualise the protein loading during western blot analysis. The contents of manganese superoxide dismutase (MnSOD) and copper/zinc superoxide dismutase (Cu/ZnSOD) were analysed by using rabbit polyclonal antibodies obtained from Enzo scientific (Cat. no. ADI-SOD-111F and ADI-SOD-100F respectively). The content of catalase was analysed using a mouse monoclonal anti-catalase antibody obtained from Sigma (Cat. no C0979). Bands were visualised and analysed using a Biorad Chemi-Doc System (Bio-Rad, Hercules, CA).

### 2.5 Statistics

Data are presented as mean+SE of values for 6–8 wells for each experiment. Data were initially analysed by analysis of variance followed by modified Student's *t* test. Data were considered significant at *p*<0.05.

## 3 Results

### 3.1 Cell morphology

**Fig. 1:**
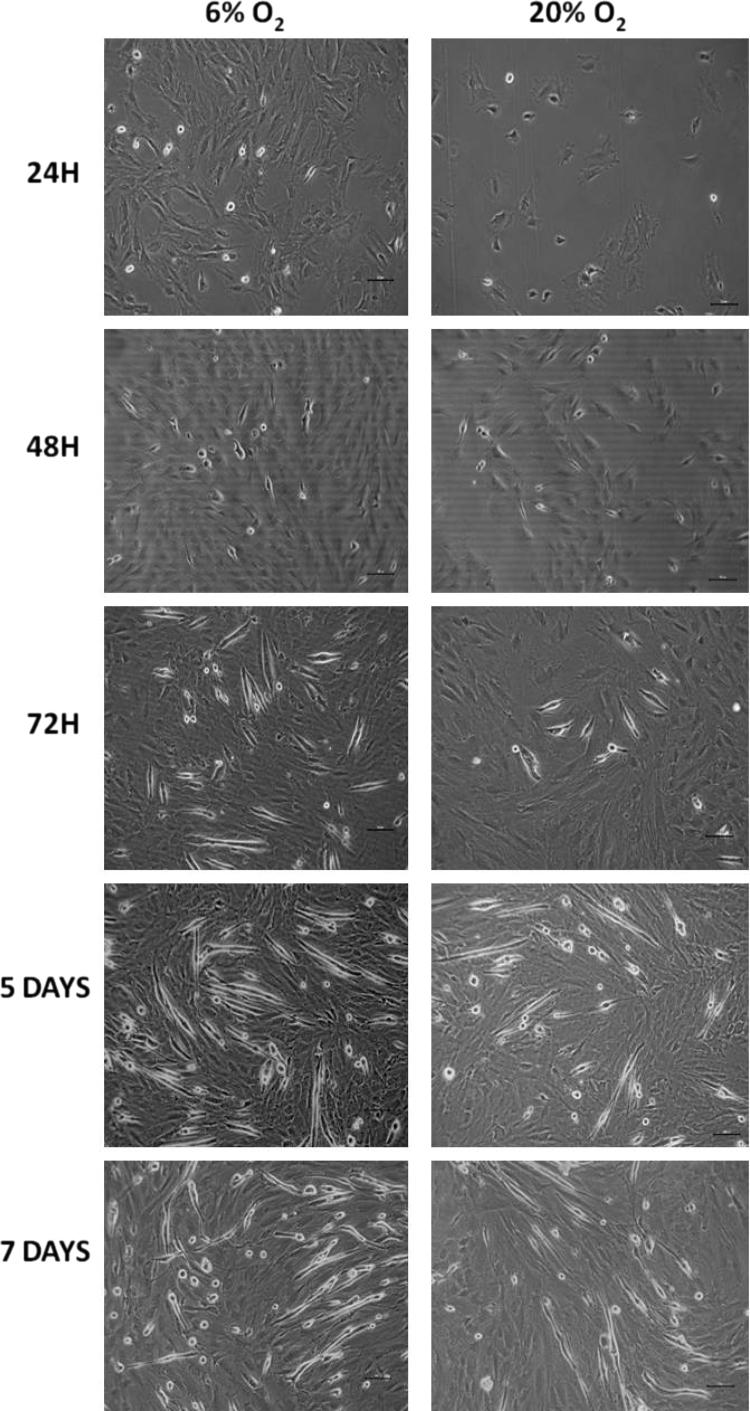
Example images of primary skeletal muscle cells from wild-type mice grown at 6% and 20% O_2_. Myoblasts were initially plated at 1×10^4^ and cultured in 20% FBS and after 2 days in culture the media was changed to 2% horse serum to promote differentiation.

Myoblasts grown either in 6% or 20% oxygen proliferated in culture and, following addition of differentiation medium, formed myotubes. There were some variations in the efficiency of proliferation and fusion; proliferation of satellite cells was clearly increased when cells were grown in 6% O_2_ compared with cells grown in 20% O_2_ (Fig. 1). Analyses of RONS activities in myoblasts and myotubes were undertaken at an approximately equivalent stage of maturation rather than at precisely the same time point following differentiation.

### 3.2 Effect of a 20% or 6% O_2_ environment on DHE, DCF and DAF-FM fluorescence in myoblasts

**Fig. 2:**
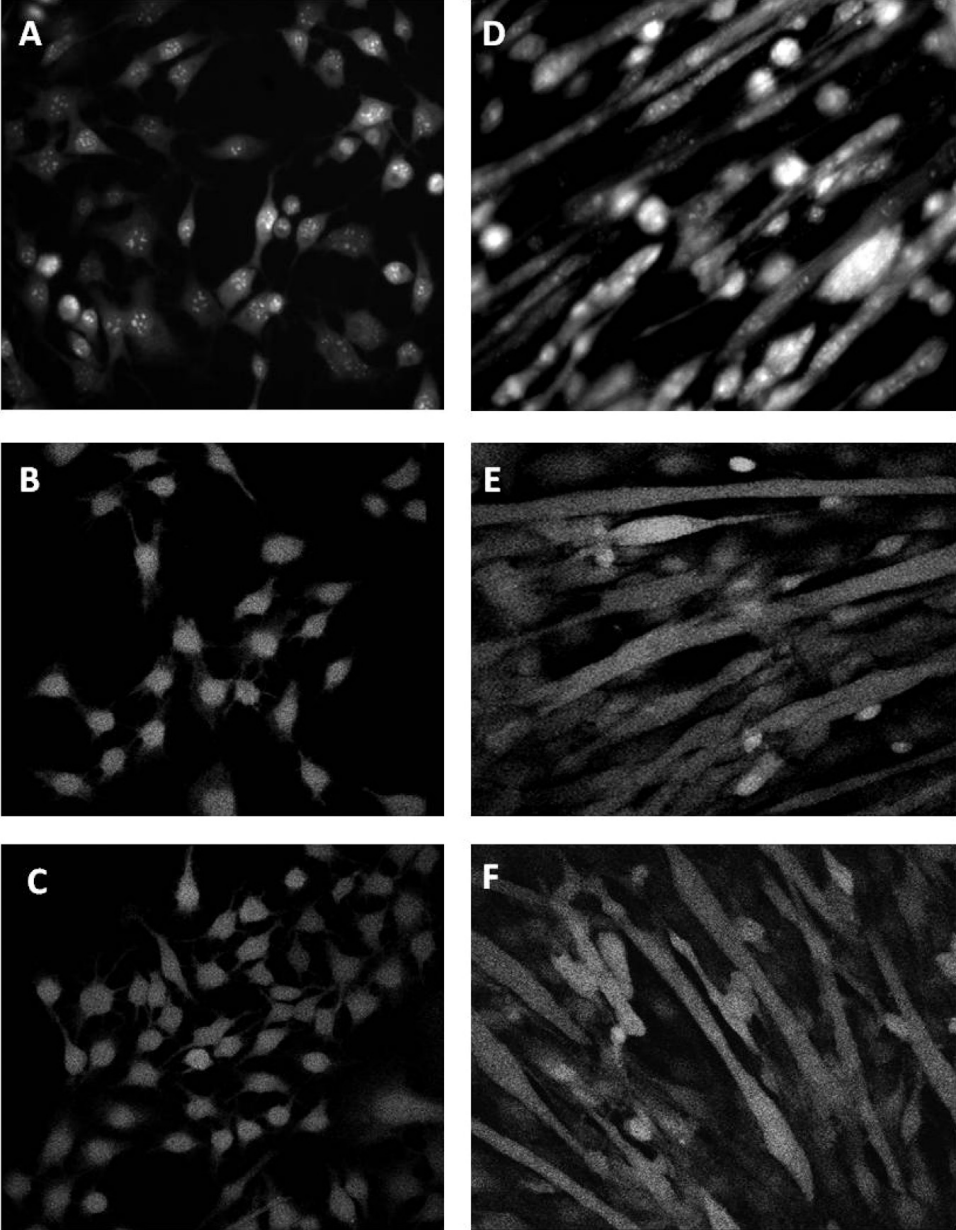
Example fluorescent images of myoblasts and myotubes from wild-type mice grown at 20% O_2_. (A) Myoblasts loaded with DHE (B) Myoblasts loaded with DCFH-DA and (C) Myoblasts loaded with DAF-FM-DA. (D) Myotubes loaded with DHE (E) Myotubes loaded with DCFH-DA and (F) Myotubes loaded with DAF-FM-DA.

**Fig. 3:**
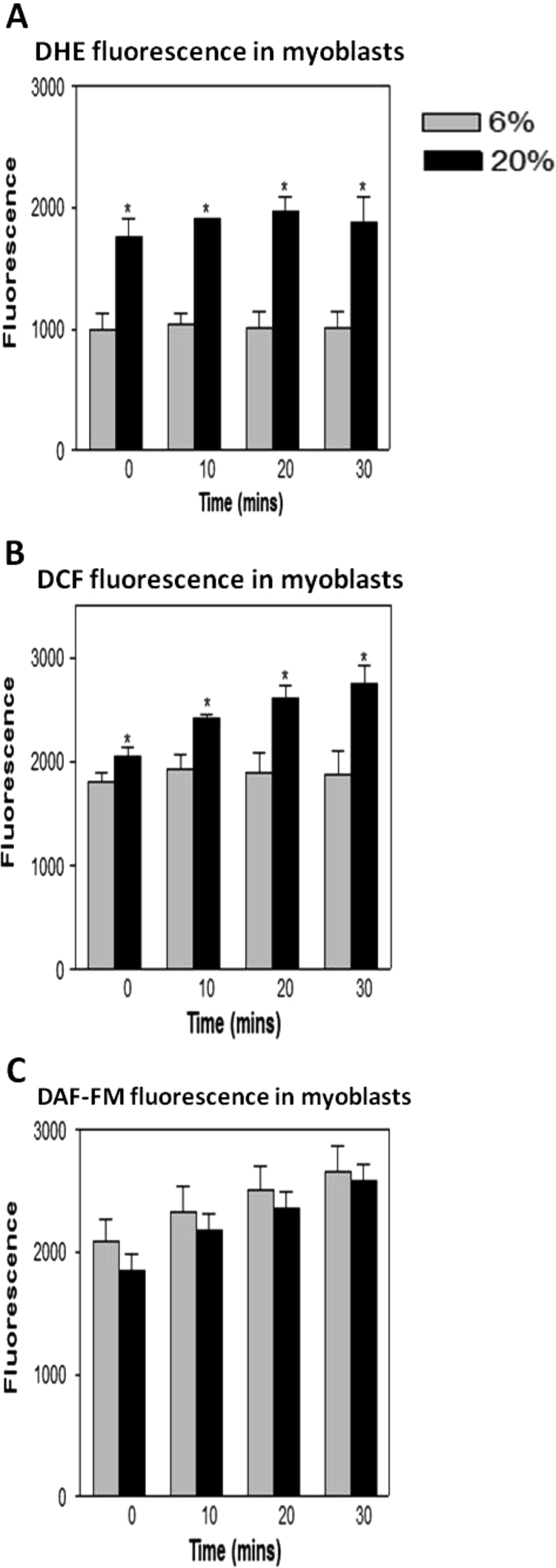
Changes in (A) DHE (B) DCF and (C) DAF-FM fluorescence in myoblasts grown in 6% or 20% O_2_ environments. Values presented as mean±s.e.m. *P<0.05.

DHE, DCF and DAF-FM fluorescence was measured from 4–6 myoblasts in each culture well (6–8 wells in total). Example images of myoblasts loaded with the 3 fluorophores are shown in Fig. 2A–C. Background measurements of fluorescence from areas of the well where myoblasts were not present were also undertaken. DHE, DCF and DAF-FM fluorescence (minus background readings) was measured over three 10-min periods for a total of 30 min. Myoblasts grown in 20% O_2_ showed a significant increase in DHE (Fig. 3A) and DCF (Fig. 3B) fluorescence at each time point compared with myoblasts grown in 6% O_2_. No significant differences were seen in DAF-FM fluorescence between myoblasts grown in 6% and myoblasts grown in 20% O_2_ concentrations (Fig. 3C).

### 3.3 Effect of a 20% or 6% O_2_ environment on DHE, DCF and DAF-FM fluorescence in myotubes

**Fig. 4:**
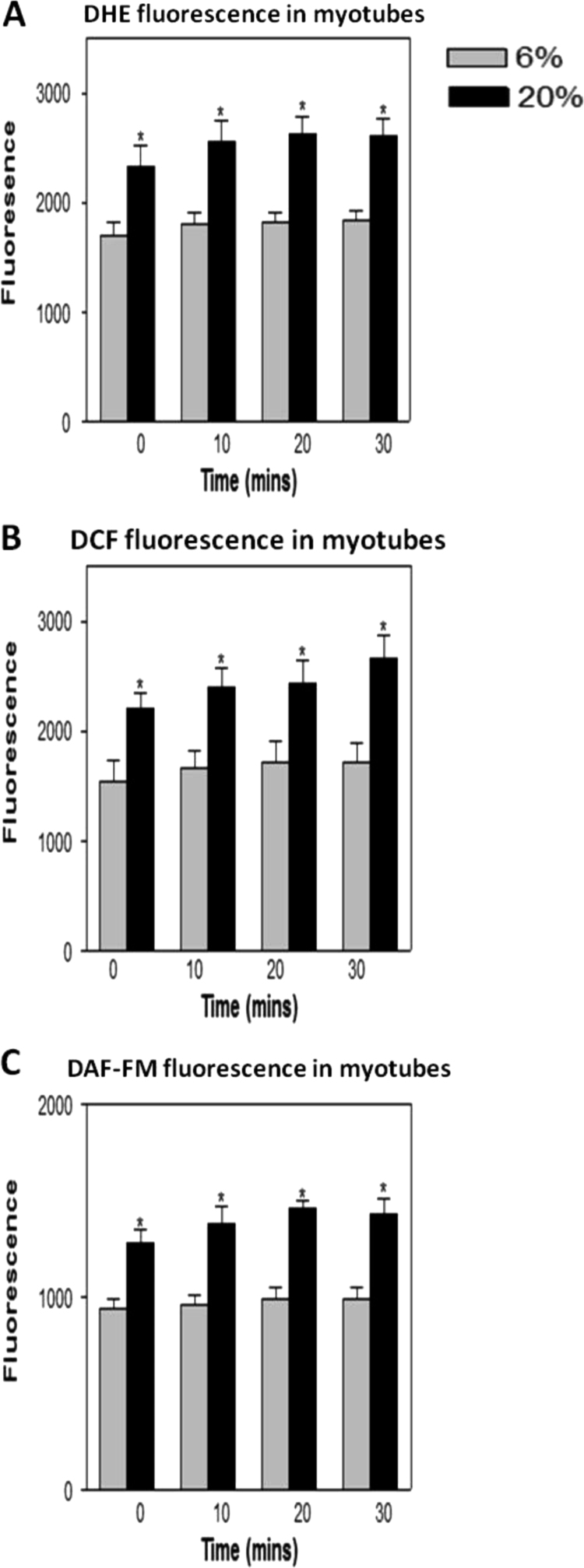
Changes in (A) DHE (B) DCF and (C) DAF-FM fluorescence in myotubes grown in 6% or 20% O_2_ environments. Values presented as mean±s.e.m. *P<0.05.

DHE oxidation and DCF and DAF-FM fluorescence were measured from at least 4 myotubes in each culture well as previously described [6]. Example images of myotubes loaded with the three fluorophores are shown in Fig. 2D–F. Background measurements of fluorescence from areas of the well where myotubes were not present were also undertaken. DHE, DCF and DAF-FM fluorescence (minus background readings) was measured over three 10-min periods for the total of 30 min. Myotubes grown at 20% O_2_ showed a significant increase at each time point in DHE oxidation and DCF and DAF-FM fluorescence in comparison with myotubes maintained in 6% O_2_ concentration (Fig. 4A–C).

### 3.4 Effect of a 20% or 6% O_2_ environment on catalase, MnSOD and Cu/ZnSOD contents in myoblasts and myotubes

**Fig. 5:**
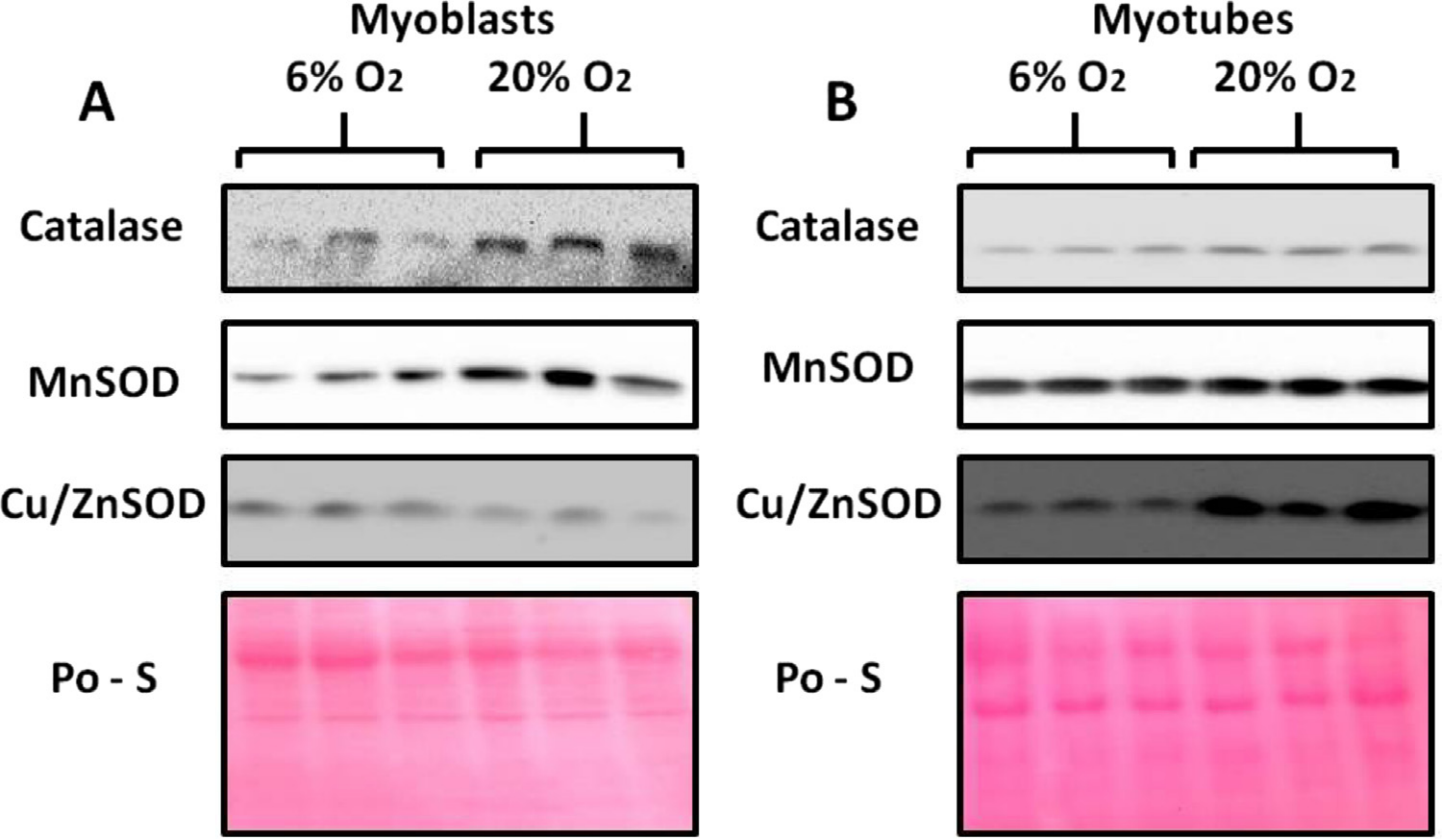
Representative western blots of catalase, MnSOD and Cu/ZnSOD in (A) myoblasts and (B) myotubes grown in 20% or 6% O_2_ environments. Ponceau S staining (Po-S) shows equal amount of protein loading.

**Fig. 6:**
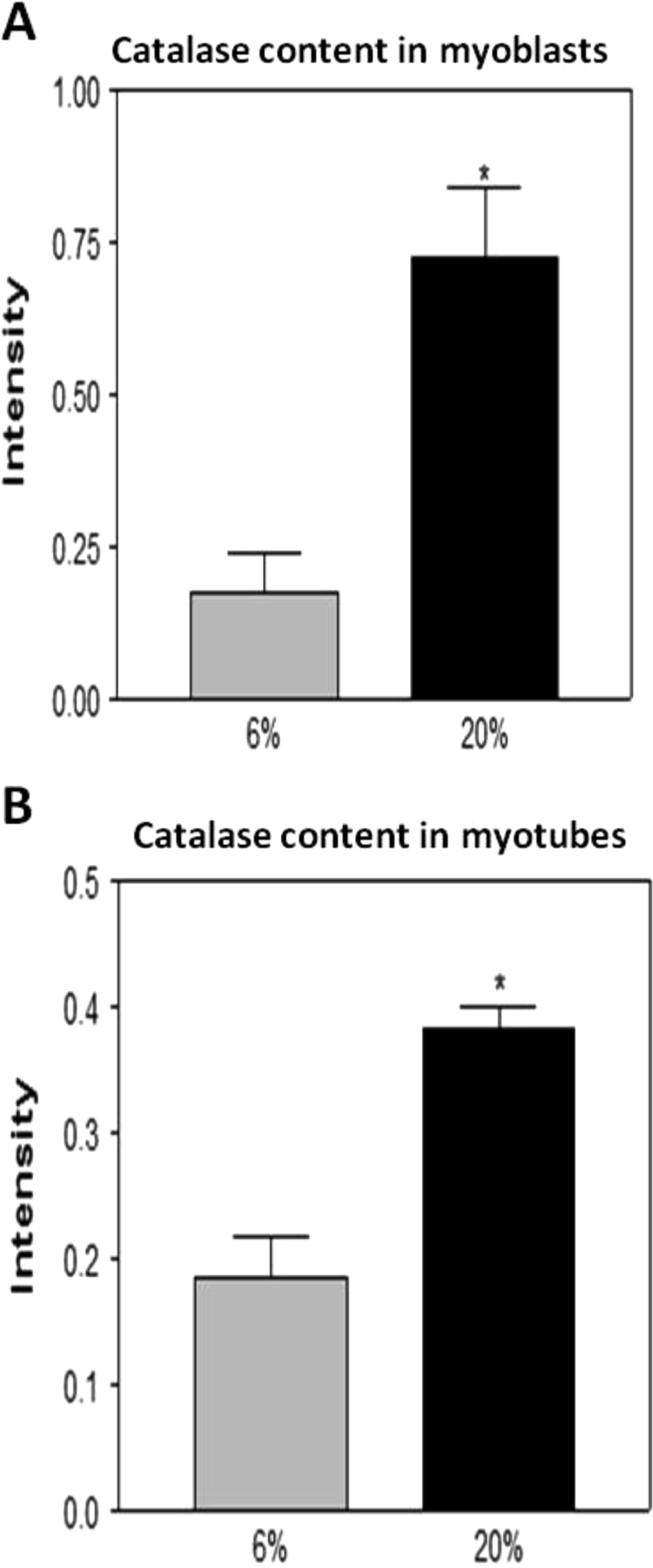
Catalase content in (A) myoblasts and (B) myotubes grown in 6% or 20% O_2_ environments. * *P*<0.05.

**Fig. 7:**
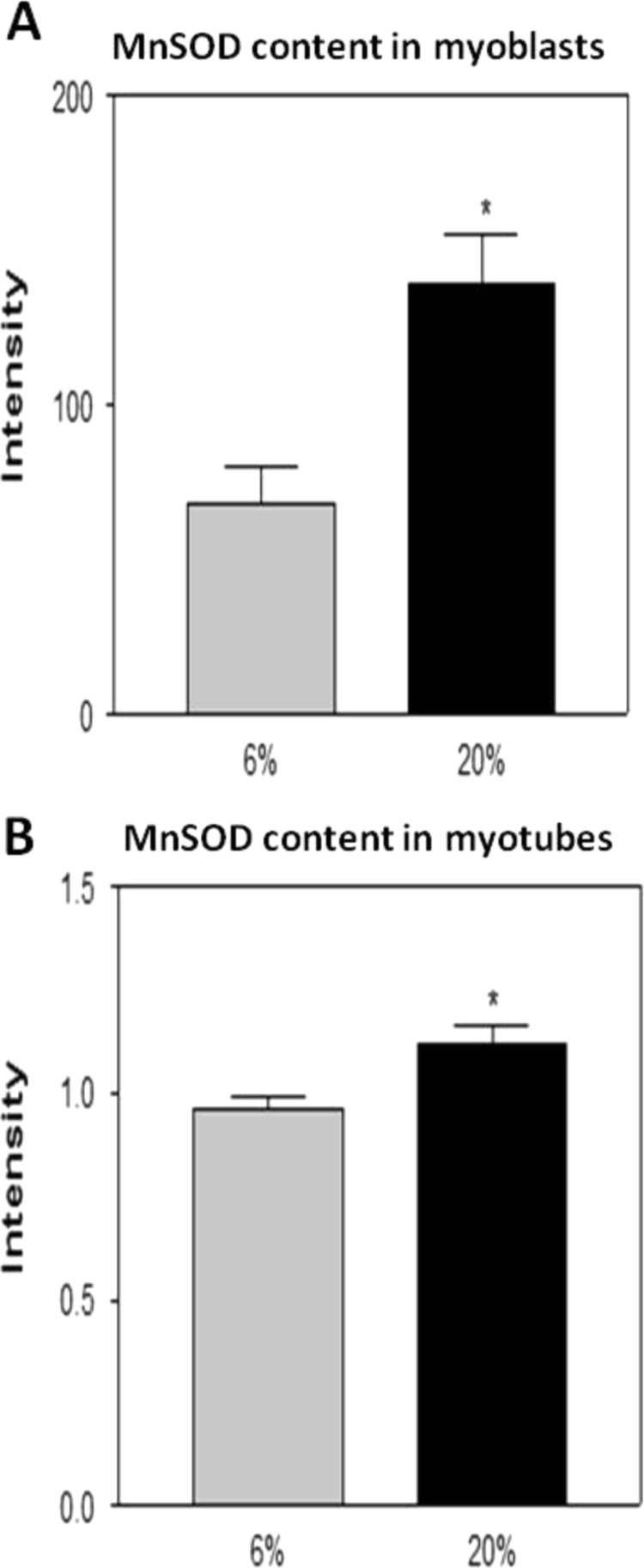
MnSOD content in (A) myoblasts and (B) myotubes grown in 6% or 20% O_2_ environments. * *P*<0.05.

**Fig. 8:**
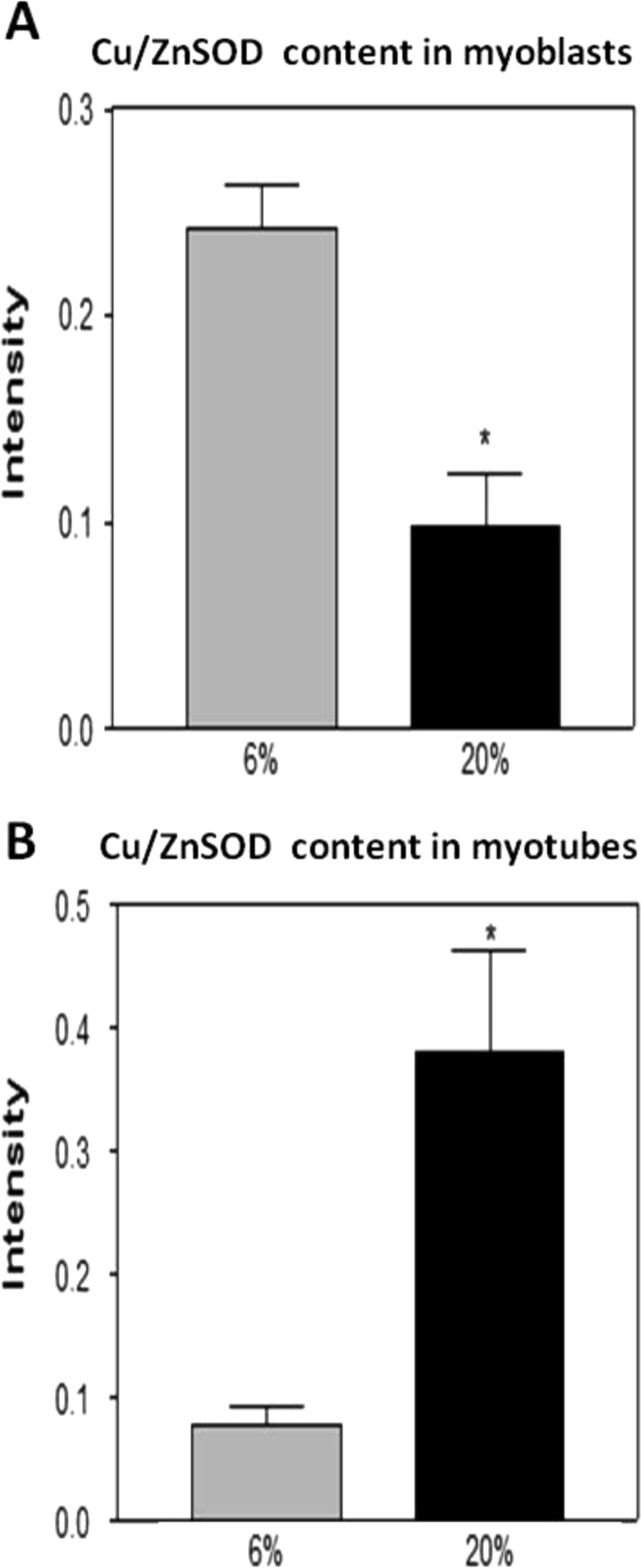
Cu/ZnSOD content in (A) myoblasts and (B) myotubes grown in 6% or 20% O_2_ environments. * *P*<0.05.

Myoblasts grown in 20% oxygen showed a significant increase in catalase (Figs. 5A and 6A) and MnSOD (Figs. 5A and 7A) contents compared with myoblasts grown in 6% O_2_. In contrast, the Cu/ZnSOD content in myoblasts grown in 20% O_2_ was significantly decreased (Figs. 5A and 8A) when compared to that at 6% O_2_. Myotubes grown at 20% O_2_ showed a significant increase in catalase (Figs. 5B and 6B), MnSOD (Figs. 5B and 7B) and Cu/ZnSOD (Figs. 5B and 8B) contents in comparison with myotubes maintained in 6% O_2_ concentration.

## 4 Discussion

In the majority of cell culture studies, cells are maintained at ambient O_2_ tension despite the fact that the physiological level *in vivo* is generally much lower. In adult skeletal muscle, the physiological tissue O_2_ levels measured by direct microelectrode analysis vary between 1.8 and 10.5%, depending on electrode placement [3], [4]. These values are well below the usual O_2_ culture conditions. In the current study, we utilised fluorescence imaging microscopy to allow monitoring of real-time changes in RONS in primary cultures using satellite cells isolated from WT mice that had been maintained at 20% or 6% oxygen environments. We hypothesised that myoblasts and myotubes grown in 20% O_2_ would have increased intracellular ROS compared with cells grown in 6% O_2_ and that the increased formation of ROS at 20% O_2_ would be associated with reduced skeletal muscle proliferation and differentiation.

Previous studies have demonstrated enhanced proliferation in physiologic O_2_ when compared to 20% O_2_ incubator conditions for several different types of cells including CNS-derived multipotent stem cells [9], neural crest stem cells [10] and marrow-derived mesenchymal stem cells [11]. In skeletal muscle, enhanced proliferation in physiological O_2_ concentration has been reported in human skeletal muscle precursor cells derived from elderly donors [12], as well as in primary rat myoblasts [13] and in primary mouse satellite cells [3], [14], [15] and this enhanced proliferation in low O_2_ conditions is associated with the up-regulation of multiple MyoD family myogenic regulatory factors (MRFs) [3]. In our study, we observed that proliferation of satellite cells was increased in physiological O_2_ which is in agreement with previously published data. The high levels of O_2_ used in the laboratory tissue culture conditions (20%) are never encountered by cells *in vivo*. A likely consequence of hyperoxia is elevated oxidative activity from RONS and this increased oxidative activity may in turn selectively drive or inhibit a variety of transcriptional pathways [16]. Environmental O_2_ concentrations have been reported to modify myogenesis *in vitro* though processes involving ROS generation [3], [5]. For example, tumour necrosis factor-alpha (TNF-α), is induced by ROS and is a potent inhibitor of skeletal muscle differentiation [17], [18]. In addition, Hansen et al. have shown that excessive ROS inhibited myogenesis in muscle cultures, while treatment of the muscle cells with the ROS trapping agent phenyl-N-tert-butylnitrone (PBN) produced a more reductive cellular redox potential and enhanced myoblast differentiation [5]. In previous collaborative studies, we have also demonstrated that myoblasts derived from glutathione peroxidase 1 knockout (*Gpx*1^−/−^) mice had increased ROS activities [6] and decreased proliferation and increased apoptosis compared with wild-type cells and differentiated poorly with many residual mononuclear cells and the formation of only few, immature myotubes [19].

To assess whether the decreased proliferation of muscle cells observed by us and others in 20% O_2_ is associated with an increase in ROS generation, intracellular ROS were measured at different stages of myogenesis using CM-DCFH-DA, DHE and DAF-FM-DA fluorescent probes. Based on our previous published work, use of these probes allows the assessment of specific ROS in single cells and we have extensively used these probes to detect ROS activities in skeletal muscle myotubes and single muscle fibres [6], [20], [21], [22], [23]. Assessment of ethidium fluorescence (E^+^) following DHE loading as a measure of superoxide anion radical in cellular compartments has been criticised and recent studies have identified 2-hydroxyethidium (2-OH-E^+^) as a specific product of the reaction of DHE with superoxide [24]. We have previously evaluated the use of DHE oxidation and showed that E^+^ and 2-OH-E^+^ followed the same pattern of change in resting and contracted fibres from wild-type mice and mice lacking Cu/ZnSOD [25]. Our previous work also demonstrated that the anticipated increase in DHE oxidation following contractile activity was completely abolished following loading of muscle fibres with the superoxide scavengers Tiron or Tempol [25]. Thus, we argue that the technique used in this study, based on monitoring E^+^ fluorescence from mature fibres, is capable of detecting changes in superoxide production at rest. We also used CM-DCFH as the ROS-sensitive probe in these studies. CM-DCFH is widely used to provide a general assessment of RONS in cells but is also widely acknowledged to be non-specific and subject to artefact due to its high sensitivity to photo-oxidation and autoxidation. We have previously described the technical approach used here to minimise photo-oxidation and autoxidation in isolated FDB fibres [26]. Murrant and Reid [27] have reported that DCFH in skeletal muscle could be oxidised by H_2_O_2_, hydroxyl radical, NO, and peroxynitrite, however the data presented here do not allow assessment of which specific species oxidised the DCFH. NO availability was examined using the NO-specific probe DAF-FM essentially as described by Pye et al. [22]. DAF-FM-DA readily diffuses into cells and within the cytoplasm releases DAF-FM by the action of intracellular esterases. We have previously shown that treatment of isolated fibres with the NO synthase inhibitors l-NAME or l-NMMA reduced the increase in DAF-FM fluorescence observed in response to contractions [22]. However, treatment of fibres with Tiron also reduced the increase in fluorescence observed during contractions [22] suggesting that superoxide, or more probably peroxynitrite, contributes to the fluorescence observed. Thus this technique can be used to examine NO generation in quiescent skeletal muscle cells in real time, although peroxynitrite and other reactive nitrogen species may potentially contribute to the fluorescence values observed.

Our data demonstrated that myoblasts and myotubes grown in 20% O_2_ showed a significant increase in DCF fluorescence and DHE oxidation compared with myoblasts grown at 6% O_2_. The catalase and MnSOD contents were also increased in myoblasts that were maintained in 20% O_2_ whereas the Cu/ZnSOD content was significantly decreased. Duguez et al. [15] have demonstrated that inhibition of proliferation of H-2K myoblasts in 20% O_2_ versus 5% O_2_ is associated with greater mitochondrial activity which in our case, may explain the increased MnSOD content. Furthermore, hydrogen peroxide can freely diffuse across the mitochondrial membrane to oxidise cytosolic DCFH and this may explain the increase in DCF fluorescence that we have observed, as well as the increase in the catalase content. Interestingly the content of Cu/ZnSOD was significantly decreased in myoblasts grown in 20% O_2_ compared with myoblasts grown at 6% O_2_. This might also explain the increase in DHE oxidation that is evident in myoblasts maintained in 20% O_2_ during proliferation.

Myotubes grown in 20% O_2_ showed a significant increase in DCF fluorescence and DHE oxidation compared with myotubes grown in 6% O_2_. The catalase, MnSOD and Cu/ZnSOD contents were increased in myotubes that were maintained in 20% O_2_. It is possible that the high O_2_ usually used in tissue culture laboratories may lead to cumulative RONS damage, such as that associated with cellular ageing, rather than reproducing the *in vivo* microenvironment in which normal muscle development and regeneration occurs. It is well documented that during ageing, the intracellular redox status shifts towards a more oxidised environment, permitting local persistence of ROS [21] as well as increased oxidative damage [28], [29], [30] and Konigsberg et al. [14] have shown that mpc's isolated from old mice show a reduced proliferation rate compared with mpc's isolated from young mice even when grown at low O_2_ concentrations. Therefore, higher O_2_ concentrations may result in a decreased proliferative cell potential that contributes to cell dysfunction and possibly to premature ageing.

It has been proposed that changes in nitric oxide (NO) production facilitate fusion of cultured myoblasts to myotubes [31]. The neuronal isoform of nitric oxide synthase (nNOS) is highly expressed in skeletal muscle [32]. We attempted to detect nNOS in our primary myoblast cultures however the level of nNOS present was undetectable regardless of the environmental O_2_ concentration suggesting that nNOS is expressed in very low levels in skeletal muscle myoblasts (data not shown). This may explain why we did not observe any significant differences in DAF-FM fluorescence between myoblasts grown in 6% and myoblasts grown in 20% oxygen concentrations. Similar to our data, previously published data by Abdelmoity et al. have demonstrated that very little if any nNOS was detected in C2C12 myoblasts during proliferation [33]. However, the authors demonstrated and nNOS content increased during differentiation of C2C12 myoblasts. We did not observe any increases in the content of nNOS present in differentiating primary myoblasts regardless of the environmental O_2_ concentration (data not shown) suggesting that the content of nNOS is different between primary skeletal muscle myotubes and C2C12 myotubes [33]. Since both proliferation and differentiation were enhanced in cells grown in 6% O_2_ it is possible that myotubes grown in 20% O_2_ had a slower rate of fusion that could explain the increase in DAF-FM fluorescence in myotubes maintained at 20% O_2_ compared with myotubes maintained at 6% O_2_. As mentioned above, it is also possible that peroxynitrite and other reactive nitrogen species may have contributed to the increased DAF-FM fluorescence values observed in myotubes grown in 20% O_2_.

In conclusion, these data suggest that superoxide and/or hydrogen peroxide (but not nitric oxide) reduce myogenesis in primary myoblasts. The data indicate that myoblast proliferation and differentiation may be influenced by changes in environmental O_2_ concentration via a process linked to ROS. These data highlight the importance of considering O_2_ concentration as a critical factor in cell culture studies especially with respect to the significance of redox signalling during myogenesis.
